# Fucoidan Supplementation Improves Exercise Performance and Exhibits Anti-Fatigue Action in Mice

**DOI:** 10.3390/nu7010239

**Published:** 2014-12-31

**Authors:** Yi-Ming Chen, Yi-Hsin Tsai, Tsung-Yu Tsai, Yen-Shuo Chiu, Li Wei, Wen-Chyuan Chen, Chi-Chang Huang

**Affiliations:** 1 Graduate Institute of Sports Science, National Taiwan Sport University, Taoyuan 33301, Taiwan; E-Mails: 1021302@ntsu.edu.tw (Y.-M.C.); 1020209@ntsu.edu.tw (Y.-H.T.); yschiu12369@yahoo.com.tw (Y.-S.C.); 2 Department of Food Science, Fu Jen Catholic University, Taipei 24205, Taiwan; E-Mail: tytsai@mail.fju.edu.tw; 3 Department of Orthopedic Surgery, Taipei Medical University-Shuang Ho Hospital, New Taipei City 23561, Taiwan; 4 Department of Neurosurgery, Taipei Medical University-WanFang Hospital, Taipei City 11696, Taiwan; E-Mail: nsweili@gmail.com; 5 Center for General Education, Chang Gung University of Science and Technology, Taoyuan 33301, Taiwan

**Keywords:** brown seaweed extract, exercise performance, forelimb grip strength

## Abstract

Fucoidan (FCD) is a well-known bioactive constituent of seaweed extract that possess a wide spectrum of activities in biological systems, including anti-cancer, anti-inflammation and modulation of immune systems. However, evidence on the effects of FCD on exercise performance and physical fatigue is limited. Therefore, we investigated the potential beneficial effects of FCD on ergogenic and anti-fatigue functions following physiological challenge. Male ICR mice from three groups (*n* = 8 per group) were orally administered FCD for 21 days at 0, 310 and 620 mg/kg/day, which were, respectively, designated the vehicle, FCD-1X and FCD-2X groups. The results indicated that the FCD supplementations increased the grip strength (*p* = 0.0002) and endurance swimming time (*p* = 0.0195) in a dose-depend manner. FCD treatments also produced dose-dependent decreases in serum levels of lactate (*p* < 0.0001) and ammonia (*p* = 0.0025), and also an increase in glucose level (*p* < 0.0001) after the 15-min swimming test. In addition, FCD supplementation had few subchronic toxic effects. Therefore, we suggest that long-term supplementation with FCD can have a wide spectrum of bioactivities on health promotion, performance improvement and anti-fatigue.

## 1. Introduction

Fucoidan (FCD) a well-known bioactive phytocompound of brown seaweed, edible and economic brown algae used in food, feed and energy industries. FCD is a sulfated polysaccharide that contains substantial percentages of L-fucose and sulfate ester groups [1,2]. Research articles on FCD have escalated dramatically in the last 20 years. While PubMed shows 179 FCD publications in the decade from 1984 to 1993, there were 370 from 1994 to 2003, and 541 from 2004 to 2013. Numerous investigations revealed that FCD exhibits various biological effects, such as antimetastatic activity by blocking the interactions between cancer cells and the basement membrane [3]; induction of cancer cells apoptosis [4]; anticoagulant activity [5], antii-nflammation [6,7]; antimicrobial activity [8]; and antioxidation [9,10]. These findings have stimulated an explosion of investigations on FCD, its bioactivities and its possible role in human health.

Fatigue is a symptom, which is defined as organism physiological fatigue, where operation cannot be maintained and the organs do not remain favorable working conditions. However, fatigue is always difficult to define. This is because of the unique intrinsic properties and anatomic features of individual muscles [11]. Exercise leads to changes in metabolism; energy provision; and cardiovascular, respiratory, thermoregulatory, and hormonal responses [12]. As a result of demand exceeding capacity in one or more systems, either directly in the active muscles (peripheral fatigue) or the central nervous system (central fatigue), causes fatigue and the termination of exercise [13]. It is well documented that oxidative stress, energy source depletion, and excess metabolite accumulation are involved in the occurrence of physical fatigue [14,15,16]. Studies have also shown that antioxidants supplementation could prolong exercise performance, reduce metabolite production, and reduce physical fatigue [16,17]. Many studies show that FCD possess significant antioxidant activity, both *in vitro* and *in vivo*. FCD has great potential for preventing free radical-mediated degradation of DNA in human umbilical vein endothelial cells [18] and protect against chemical-induced oxidative damage in mice [19]. However, according to a search of the PubMed database, there are still relatively few studies that directly address the possible ergogenic or anti-fatigue function of FCD. Therefore, the objective of this study was to evaluate the effects of FCD on exercise performances and fatigue-associated biochemical indices according to our previous reports [17,20].

## 2. Experimental Section

### 2.1. Materials, Animals, and Experiment Design

Fucoidan isolated from *Laminaria japonica* and was purchased from Wel-Bloom Bio-Tech Corporation (Taipei City, Taiwan). The certificate of analysis for the test material is provided as a Supplementary document. Male ICR strain mice (6 weeks old) with specific pathogen free condition were purchased from BioLASCO (Yi-Lan, Taiwan). All animals were provided with a standard laboratory diet (No. 5001; PMI Nutrition International, Brentwood, MO, USA) and distilled water *ad libitum*, and housed at 12-h light/12-h dark cycle at room temperature (22 °C ± 1 °C) and 50%–60% humidity. The Institutional Animal Care and Use Committee (IACUC) of National Taiwan Sport University (NTSU) inspected all animal experiments, and this study conformed to the guidelines of protocol IACUC-10206 approved by the IACUC ethics committee.

In this study, the dose of FCD designed for humans is 1.5 g per day. The mouse FCD dose (0.31 g/kg) used here was converted from a human equivalent dose (HED) based on body surface area by the following formula from the US Food and Drug Administration: assuming a human weight of 60 kg, the HED for 1.5 (g) ÷ 60 (kg) = 0.025 × 12.3 = a mouse dose of 0.31 g/kg; the conversion coefficient 12.3 was used to account for differences in body surface area between a mouse and a human as described in our recent study [21].

Twenty-four mice were randomly assigned to 3 groups (8 mice/group) for FCD treatments: (1) vehicle; (2) 0.31 g/kg FCD (FCD-1X); and (3) 0.62 g/kg FCD (FCD-2X). The vehicle group received the same volume of solution equivalent to individual BW and all treatments were given orally to each mouse for a 21-day duration.

### 2.2. Forelimb Grip Strength Test

A low-force testing system (Model-RX-5, Aikoh Engineering, Nagoya, Japan) was used to measure the forelimb grip strength of mice undergoing vehicle or FCD treatments. The detailed procedures were described in our previous report [15].

### 2.3. Swimming Exercise Performance Test

Swim to exhaustion exercise test involved constant loads corresponding to 5% of body weight to evaluate the endurance time as described in our previous study [17]. The swimming endurance time of each mouse was recorded from beginning to exhaustion, which was determined by observing loss of coordinated movements and failure to return to the surface within 7 s.

### 2.4. Determination of Fatigue-Associated Biochemical Variables

Effects of FCD supplementation on fatigue-associated biochemical indices were evaluated post-exercise as our previous reports [15,17,20,22]. At 1 h after the FCD supplementation, all animals underwent a 15-min swim test without weight loading. After a 15-min swim exercise, blood sample was immediately collected and centrifuged at 1500× *g* and 4 °C for 10 min for serum separation. Serum lactate, ammonia, glucose and CK levels were determined using an autoanalyzer (Hitachi 7060, Hitachi, Tokyo, Japan).

### 2.5. Clinical Biochemical Profiles

At the end of the experimental period, all mice were finally sacrificed with 95% CO_2_ asphyxiation, and blood was immediately collected at rest. Serum was collected by centrifugation and the clinical biochemical variables including AST (aspartate aminotransferase), ALT (alanine), ALP (alkaline phosphatase), LDH (lactate dehydrogenase), CK (creatine kinase), albumin, TBIL (total bilirubin), TP (total protein), BUN (blood urea nitrogen), creatinine, UA (uric acid), TC (aminotransferasetotal cholesterol), TG (triacylglycerol) and glucose were measured using an autoanalyzer (Hitachi 7060).

### 2.6. Histological Staining of Tissues

Target organs were carefully removed, minced and fixed in 10% formalin after sacrifice. All tissues were then embedded in paraffin and cut into 4-μm thick slices for morphological and pathological evaluations. Tissue sections were stained with hematoxylin and eosin (H & E) and examined using a light microscope equipped with a CCD camera (BX-51, Olympus, Tokyo, Japan) by a veterinary pathologist.

### 2.7. Statistical Analysis

All data are expressed as mean ± standard error of the mean (SEM) for *n* = 8 mice per group. Statistical differences among groups were analyzed by a one-way analysis of variance (ANOVA) and the Cochran-Armitage test for the dose-effect trend analysis with SAS ver. 9.0 (SAS Institute, Cary, NC, USA). *p* values of <0.05 were considered statistically significant.

## 3. Results and Discussion

### 3.1. Effects of FCD on Forelimb Grip Strength

**Figure 1:**
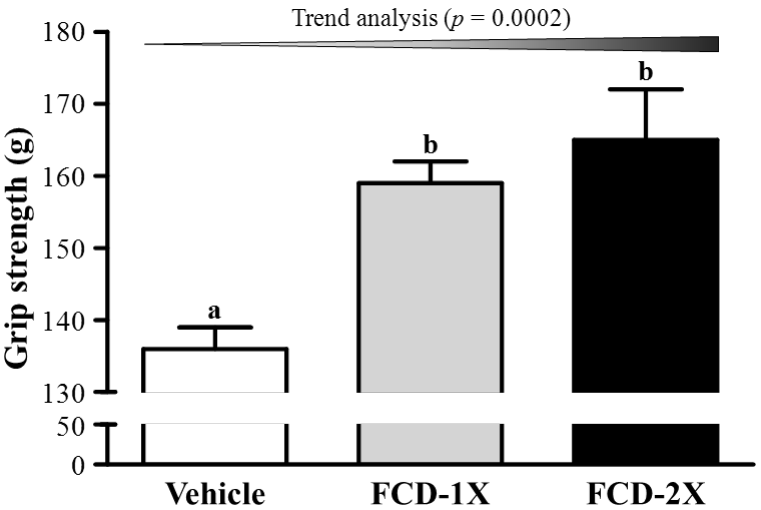
Effect of FCD (Fucoidan) supplementation on forelimb grip strength. Data are presented as the mean ± SEM of 8 mice in each group. One-way ANOVA was used for the analysis. Different letters (a, b) indicate a significant difference at *p* < 0.05. Low-dose (FCD-1X) and high-dose (FCD-2X) FCD at 310 and 620 mg/kg/day.

The forelimb grip strength values in the vehicle, FCD-1X and FCD-2X groups were 136, 159 and 165 g, respectively (Figure 1). The forelimb grip strength of the FCD-1X and FCD-2X groups were 1.17- (*p* = 0.0074) and 1.21-fold (*p* = 0.0013), respectively; significantly higher than those of the vehicle group. In the trend analysis, absolute forelimb grip strength dose-dependently increased with the FCD dose (*p* = 0.0002). In general, programmed exercise training is required to increase grip strength [21]; however, the results indicated that FCD supplementation benefited grip strength even though test animals did not undergo a training intervention. Thus, long-term FCD supplementation can benefit grip strength when no training protocol is implemented. Our previous reports have shown that seven to twenty-one days of supplementation with plant extracts, resveratrol, ethanolic extract of deer antler, or long-term supplementation with ergogenic aids, such as whey protein, improves the grip strength of untrained animals [17,20,21,22]. Thus, FCD, a substance of brown algae origin used as an ingredient in some health food, may be an alternative supplement for promoting body strength under an untrained condition or in a programmed-training protocol.

### 3.2. Effects of FCD on Exercise Performance in a Weight-Loaded Swimming Test

**Figure 2:**
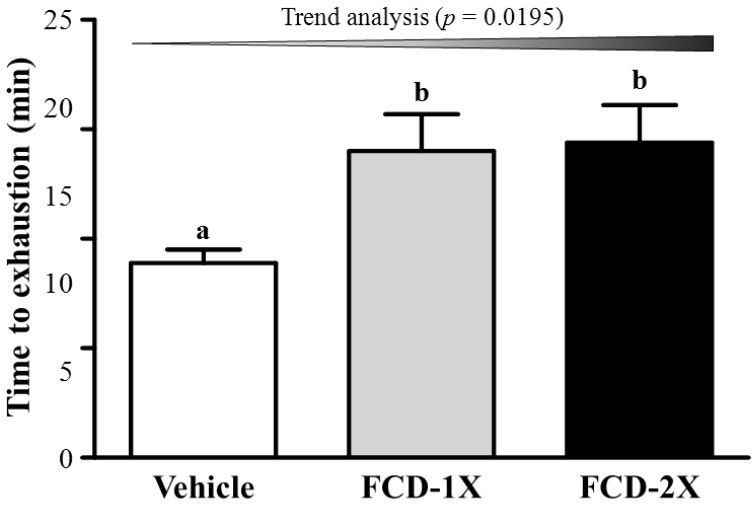
Effect of FCD (Fucoidan) supplementation on swimming exercise performance. Mice were pretreated with the vehicle, FCD-1X, FCD-2X of FCD for 21 days and, then 1 h later performed an exhaustive swimming exercise with a load equivalent to 5% of the mouse’s body weight attached to its tail. Data represent the mean ± SEM (*n* = 8 mice). One-way ANOVA was used for the analysis. Different letters (a, b) indicate a significant difference at *p* < 0.05. Low-dose (FCD-1X) and high-dose (FCD-2X) FCD at 310 and 620 mg/kg/day.

Energy metabolism during muscular activity determines the level of physiological fatigue [23]. Exercise endurance is an important index in evaluating anti-fatigue treatment. As shown in Figure 2, endurance swimming time in the vehicle, FCD-1X, and FCD-2X groups were 8.0, 14.9 and 14.4 min, respectively. The exhaustive swimming time of the FCD-1X and FCD-2X groups were 1.58- (*p* = 0.0455) and 1.63-fold (*p* = 0.0306), respectively; significantly longer than those of the vehicle group. In the trend analysis, endurance swimming time dose-dependently increased with the FCD dose (*p* = 0.0195). Based on these results, we suggest that FCD improves endurance performance in the absence of training. Therefore, further investigation is also required to elucidate the effects of long-term FCD supplementation combined with exercise training on endurance performance.

### 3.3. Effect of FCD Supplementation on Serum Lactate, Ammonia, Glucose and CK Levels after Acute Exercise Challenge

**Figure 3:**
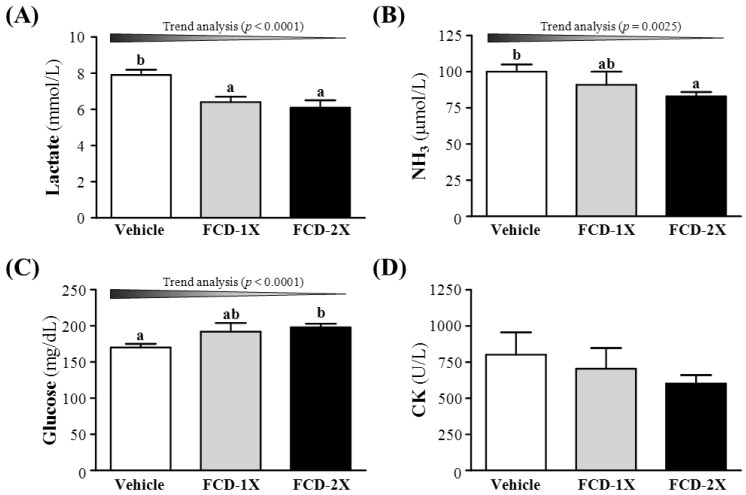
Effects of FCD (Fucoidan) supplementation on serum levels of lactate (**A**); ammonia (**B**); glucose (**C**); and CK (**D**) after an acute exercise challenge. Data represent the mean ± SEM of eight mice in each group. Columns with different letters (a, b) differ significantly, *p* < 0.05 by a one-way ANOVA. Low-dose (FCD-1X) and high-dose (FCD-2X) FCD at 310 and 620 mg/kg/day.

Post-exercise induced muscle fatigue can be evaluated by important biochemical indicators, including lactate, ammonia, glucose, and creatine kinase (CK) levels, after exercise [24,25]. Lactate accumulates when cellular glycolysis exceeds the aerobic metabolic capacity. When lactic acid concentration increases, there is a high level of hydrogen ions accumulation, and leads to fatigue due to acidification [26,27]. Therefore, lactic acid was related in the exercise intensity, glycogen storage conditions and fatigue. As shown in the Figure 3A, respective lactate levels in the vehicle, FCD-1X, and FCD-2X groups were 7.9 ± 0.3, 6.4 ± 0.3, and 6.1 ± 0.4 mmol/L; the lactate levels of the mice that received FCD-1X and FCD-2X supplementation were 18.5% (*p* = 0.0032) and 22.5% (*p* = 0.0006), respectively; significantly lower than those of mice that received the vehicle treatment. In the trend analysis, serum lactate levels dose-dependently decreased with the FCD dose (*p* < 0.0001). After acute exercise, the way of relaxing was significantly affected by blood lactate clearance rate. Approximately 75% of the total amount of lactate produced is used for oxidative production of energy in the exercising body, and it could be utilized for the *de novo* synthesis of glucose in the liver [28]. In the present study, FCD supplementation could decrease blood lactate levels and increase the glucose concentration after acute exercise challenge. Therefore, we suggest that FCD supplementation may have potentiation for the removal and utilization of blood lactate after exercise.

Ammonia, another important metabolite, accumulates with highly intensive long-lasting exercise and can be reduced by herbal supplementation [23]. During energy metabolism for exercise, ammonia is generated by different sources. The immediate source of ammonia production is the purine nucleotide cycle [29], deamination of adenosine monophosphate to inosine monophosphate, and ammonia is the substantially elevated during intensive or prolonged exercise when the ATP utilization rate may exceed the production rate. The other source could be the gluconeogenesis process via deamination of several amino acids produced by proteolysis. The direction of movement of ammonia or ammonium ion depends on concentration and pH gradients between tissues, including the brain. Although exercise-induced ammonia toxicity is transient and reversible relative to disease states, it may affect continuing coordinated activity in critical regions of the central nervous system. As shown in the Figure 3B, serum ammonia levels in the vehicle, FCD-1X and FCD-2X groups were 100 ± 5, 91 ± 9 and 83 ± 3 μmol/L, respectively. Compared with the vehicle group, serum ammonia level was slightly decreased by 17.7% (*p* = 0.0544) in the FCD-2X group.

Blood glucose level is an important index for performance maintenance during exercise [15,17,20]. Serum glucose levels in the vehicle, FCD-1X, and FCD-2X groups were 170 ± 5, 192 ± 12, and 198 ± 5 mg/dL, respectively. Values of the FCD-2X group were 1.16-fold (*p* = 0.0246); significantly higher than that of the vehicle group (Figure 3C). The trend analysis also showed that serum glucose levels dose-dependently increased with the FCD dose (*p* < 0.0001). Therefore, continuous supplemented FCD for 21 days could increase energy utilization and improve exercise performance.

Serum CK level is an important clinical biomarker of muscle damage, muscular dystrophy, severe muscle breakdown, myocardial infarction, autoimmune myositides and acute renal failure. High-intensity exercise challenge could physically or chemically cause tissue damage and muscular cell necrosis [30]. The serum CK concentration was lower in normal state, while muscle tissue CK activity increased as hypoxia and the accumulation of metabolites during exercise caused by muscle cells damage resulting in decreased exercise performance [31]. Serum CK levels in the vehicle, FCD-1X and FCD-2X groups were 800, 704 and 602 mg/dL, respectively (Figure 3D). There was no significant difference among the three groups. Therefore, our study suggested the FCD supplementation for 21 days may not affect the serum CK levels post an acute exercise challenge.

Previous study showed that tumor necrosis factor (TNF) is synthesized in the mechanically when the muscle cells received the stress and probably play an important role to cause the fatigue [32]. Recent study further demonstrated that pro-inflammatory cytokines, IL-6 and TNF-α, reduces the intracellular glycogen stock and lead to fatigue [33], and FCD could down regulated the Il-6 and TNF-α levels both *in vitro* and *in vivo* [34,35,36]. In addition, FCD used in this study was isolated from *Laminaria japonica*. Previous showed that the backbone of FCD was primarily (1→3)-linked α-l-fucopyranose residues (75%) and a few (1→4)-α-l-fucopyranose linkages (25%). Moreover, the molar ratio of sulfate to fucose content plays an important role on the free-radical scavenging activity of FCD [37]. Therefore, we suggest that FCD may have the potential to develop as an ergogenic supplement partly by its anti-inflammation and antioxidant activity.

### 3.4. Subchronic Toxicity Evaluation of FCD Supplementation

Subchronic toxic evaluation of FCD supplementation was evaluated by animals’ behavior, dietary, growth, organs weight, clinical biochemistry and histopathology. The vehicle and FCD supplementation groups did not differ in daily behavior during treatment. Morphological data from experimental groups are summarized in the Table 1. There was no significant difference in initial BWs among the vehicle, FCD-1X, and FCD-2X groups. Because we observed a significant increase in the daily intake of diet and water in FCD-fed mice, the effects of FCD on the final BW, and liver, muscle and brown adipose tissue (BAT) mass gain were of primary interest. The food intake and water consumption of the FCD-2X group was significantly higher by 1.07- (*p* = 0.0009) and 1.15-fold (*p* = 0.0003), respectively, compared to the vehicle group. Consistent with the food intake data in the FCD-2X group, we found the final BW of FCD-2X group was significant higher compared to the vehicle group (Table 1). The trend analysis showed significant increases in the final BW (*p* = 0.0003) and food intake (*p* < 0.0001) with an increasing dosage of FCD supplementation. Therefore, the effect of FCD on increasing the BW was clearly dependent on food intake. In addition, the trend analysis also showed significant increases in tissues weights of the liver (*p* = 0.0073), muscles (*p* = 0.0089), and BAT (*p* = 0.0081) with an increasing dosage of FCD treatment. The relative tissue weight (%) is a measure of different tissue weights adjusted for the individual BW, and there were no significant changes in the relative liver, skeletal muscle (gastrocnemius and soleus muscles), heart, lung, kidney, epididymal fat pad (EFP) or BAT weights (%) among the vehicle, FCD-1X, and FCD-2X groups (Table 1). We also found no gross abnormalities attributed to FCD treatment when weighing organs.

**Table 1:** General characteristics of the experimental groups.

Characteristic	Vehicle	FCD-1X	FCD-2X	Trend Analysis
Initial BW (g)	31.9 ± 0.6	32.7 ± 0.4	32.8 ± 0.5	0.1167
Final BW (g)	35.1 ± 0.7 ^a^	36.3 ± 0.5 ^ab^	38.1 ± 0.6 ^b^	<0.0003 (↑)
Food intake (g/day)	6.1 ± 0.0 ^a^	6.6 ± 0.1 ^b^	6.6 ± 0.1 ^b^	<0.0001 (↑)
Water intake (mL/day)	7.6 ± 0.1 ^a^	8.5 ± 0.2 ^b^	8.8 ± 0.2 ^b^	<0.0001 (↑)
***Weight (g)***				
Liver	1.98 ± 0.08 ^a^	2.01 ± 0.06 ^ab^	2.20 ± 0.04 ^b^	0.0073 (↑)
Muscle	0.35 ± 0.01 ^a^	0.35 ± 0.01 ^ab^	0.38 ± 0.01 ^b^	0.0089 (↑)
Heart	0.21 ± 0.01	0.21 ±0.01	0.22 ± 0.01	0.8012
Lung	0.38 ± 0.03	0.39 ± 0.02	0.43 ± 0.03	0.0901
Kidney	0.62 ± 0.05	0.61 ± 0.02	0.63 ± 0.02	0.4339
EFP	0.49 ± 0.04	0.58 ± 0.07	0.56 ± 0.03	0.2890
BAT	0.159 ± 0.009 ^a^	0.167 ± 0.007 ^ab^	0.184 ± 0.006 ^b^	0.0081 (↑)
***Relative Weight (%)***				
Liver	5.64 ± 0.16	5.54 ± 0.10	5.79 ± 0.08	0.2432
Muscle	0.99 ± 0.02	0.97 ± 0.01	0.99 ± 0.01	0.9784
Heart	0.61 ± 0.03	0.59 ± 0.03	0.59 ± 0.03	0.6053
Lung	1.08 ± 0.10	1.08 ±0.07	1.13 ± 0.07	0.5161
Kidney	1.76 ± 0.12	1.68 ± 0.07	1.65 ± 0.04	0.7710
EFP	1.40 ± 0.11	1.58 ± 0.18	1.47 ± 0.09	0.5741
BAT	0.45 ± 0.02	0.46 ± 0.02	0.48 ± 0.02	0.1330

Values are the mean ± SEM for *n* = 8 mice in each group. Values in the same line with different superscripts letters (a, b) differ significantly, *p* < 0.05 by one-way ANOVA. Food efficiency ratio: BW gain (g/day)/food intake (g/day). Muscle mass includes both gastrocnemius and soleus muscles in the back part of the lower legs. BW: body weight; BAT: brown adipose tissue; EFP: epididymal fat pad; FCD: Fucoidan. Low-dose (FCD-1X) and high-dose (FCD-2X) FCD at 310 and 620 mg/kg/day. ↑, dose-dependently increased by FCD treatment.

### 3.5. Effect of FCD Supplementation on Biochemical Analyses at the End of the Experiment

**Figure 4:**
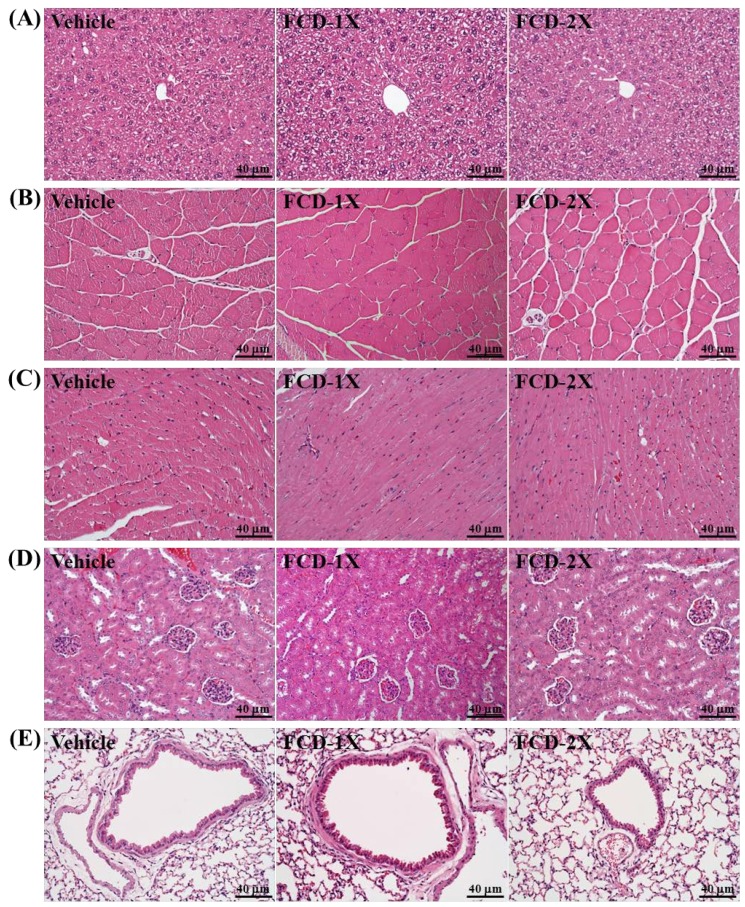
Effect of FCD (Fucoidan) supplementation on the morphology of liver (**A**); skeletal muscle (**B**); heart (**C**); kidney (**D**); and lungs (**E**) tissues. Specimens were photographed with a light microscope (BX-51, Olympus, Tokyo, Japan). (H & E stain, magnification: 200×, Scale bar, 40 μm). Low-dose (FCD-1X) and high-dose (FCD-2X) FCD at 310 and 620 mg/kg/day.

In the present study, we observed beneficial effects of FCD on the grip strength, exhaustive exercise challenge and measured other physiological effects with 21 days of FCD supplementation. We further investigated whether FCD treatments with 21 days could cause any negative effect on other biochemical markers of healthy mice. We examined the tissues- and health status-related biochemical parameters and major organs including liver, skeletal muscle, heart, kidney, and lung according to histopathological examinations in FCD-treated mice (Table 2 and Figure 4).

**Table 2:** Biochemical analysis of the FCD treatment groups at the end of the experiment.

Parameter	Vehicle	FCD-1X	FCD-2X	Trend Analysis
AST (U/L)	70 ± 5 ^b^	57 ± 3 ^a^	60 ± 4 ^ab^	0.1327
ALT (U/L)	51 ± 2	42 ± 2	45 ± 5	0.1072
ALP (U/L)	283 ± 12 ^ab^	263 ± 19 ^a^	311 ± 13 ^b^	0.1086
LDH (U/L)	328 ± 17	342 ± 26	350 ± 29	0.4820
CK (U/L)	170 ± 23 ^b^	101 ± 16 ^a^	124 ± 15 ^ab^	0.1381
Albumin (g/dL)	3.5 ± 0.0 ^a^	3.7 ± 0.0 ^b^	3.6 ± 0.0 ^ab^	0.5587
TBIL (μg/dL)	62 ± 6	58 ± 5	69 ± 4	0.2365
TP (g/dL)	5.8 ± 0.1 ^a^	6.2 ± 0.1 ^b^	6.1 ± 0.1 ^b^	0.0297 (↑)
BUN (mg/dL)	29.0 ± 1.0 ^b^	24.7 ± 0.7 ^a^	26.0 ± 0.7 ^a^	0.0320 (↓)
Creatinine (mg/dL)	0.32 ± 0.01	0.31 ± 0.01	0.32 ± 0.01	0.7530
UA (mg/dL)	1.00 ± 0.05	0.98 ± 0.11	1.11 ± 0.11	3.664
TC (mg/dL)	167 ± 8	161 ± 9	152 ± 10	0.5126
TG (mg/dL)	167 ± 17 ^c^	119 ± 12 ^b^	69 ± 8 ^a^	<0.0001 (↓)
Glucose (mg/dL)	204 ± 6	191 ± 11	195 ± 9	0.4642

All mice were sacrificed at the end of experiment and examined for serum levels of clinical biochemistry. Values are mean ± SEM for *n* = 8 mice per group. Values in the same line with different superscripts letters (a–c) differ significantly, *p* < 0.05 by one-way ANOVA. AST, aspartate aminotransferase; ALT, alanine aminotransferase; ALP, alkaline phosphatase; LDH, lactate dehydrogenase; CK, creatine kinase; TBIL, total bilirubin; TP, total protein; BUN, blood urea nitrogen; UA, uric acid; TC, total cholesterol; TG, triacylglycerol; FCD: Fucoidan. Low-dose (FCD-1X) and high-dose (FCD-2X) FCD at 310 and 620 mg/kg/day. ↑, dose-dependently increased by FCD treatment. ↓, dose-dependently decreased by FCD treatment.

Levels of biochemical indices, including ALT, LDH, TBIL, creatinine, UA, TC, and glucose, did not differ among groups (*p* > 0.05, Table 2). We found that serum AST and CK levels of the FCD-1X group were significantly 17.91% (*p =* 0.0375) and 40.42% (*p =* 0.0167) lower than those of the vehicle group. Serum albumin level of the FCD-1X group was significantly 1.04-fold (*p =* 0.0218) higher than that of the vehicle group. Therefore, the daily supplementation with FCD may have potential for tissues protection and beneficial effects following high intensive exercise. In addition, serum levels of TP, an index of nutritional status, in FCD-1X and FCD-2X groups were significantly 1.07- (*p =* 0.0027) and 1.05-fold (*p* = 0.0219) higher than that of the vehicle group. The trend analysis showed significant increases in the serum TP level (*p* = 0.0297) and food intake (*p* < 0.0001) with an increasing dosage of FCD supplementation. Therefore, the effect of FCD on increasing the TP was clearly dependent on food intake.

Serum levels of BUN, an important indicator of renal function, in the FCD-1X and FCD-2X groups were 14.78% (*p =* 0.0016) and 10.22% (*p* = 0.0207), respectively; significantly lower than that of the vehicle group. The trend analysis also showed that serum BUN levels dose-dependently decreased with the FCD dose (*p* = 0.0320). *L. japonica* is a popular marine medicinal plant in China; people use it as a traditional medicine for eliminating edema, a symptom of kidney disease. Consistent with a previous report [38], we found that FCD supplementation could benefit renal function in healthy mice.

Moreover, serum levels of TG in the FCD-1X and FCD-2X groups were 28.83% (*p* = 0.0147) and 58.40% (*p* < 0.0001), respectively; significantly lower than that of the vehicle group. Serum TG levels dose-dependently decreased with FCD supplementation, with significance on trend analysis (*p* < 0.0001). Previous study showed that water-soluble polysaccharides could decrease serum TC and TG levels by increasing fecal neutral steroids and bile acid excretion [39]. FCD is classified as a water-soluble polysaccharide that is considered a dietary fiber. Our data is consistent to a previous study that FCD could remarkably reduce the levels of blood lipids of hyperlipidemic rats [40]. Therefore, we suggest that FCD may have potential to develop as therapeutics for reducing blood lipids.

### 3.6. Effect of FCD Supplementation on Histological Examinations at the End of the Experiment

On morphological observation, the arrangement of sinusoid and hepatic cords in liver showed no changes with FCD treatment (Figure 4A). The gastrocnemius muscles exhibit polygonal myofibers of uniform shape and size without rhabdomyolysis (Figure 4B). Hypertrophy and hyperplasia were not observed in heart cardiomyocytes (Figure 4C). The structure of renal tubules and glomerulus did not differ among treatments (Figure 4D). In addition, all animals showed typical tissue architectures of lung alveoli on H & E staining (Figure 4E). In a previous study, Wistar rats of both sexes were exposed to FCD at a dose of 300 mg/kg body weight/day. No mortality or other signs of toxicity were observed during six months of observation [41]. Furthermore, our histopathological examinations revealed that FCD supplementation for 21 days yielded no adverse effects in major organs such as the liver, skeletal muscle, heart, kidney and lung. Therefore, the dose of FCD supplementation used in this study was safe.

## 4. Conclusions

Fucoidan have anti-fatigue activity by decreasing plasma lactate and ammonia levels and increasing serum glucose, thereby advantaged exercise performance in mice. In this study, we found that 21 days FCD supplementation without training significantly improved the forelimb grip strength and the swimming time to exhaustion of test animals. In biochemical indices, exercise-induced fatigue-related parameters including lactate, ammonia, glucose, were positively modulated by FCD supplementation in a dosage-dependent manner. In addition, FCD showed beneficial effects on the lipid profile, and liver and renal functions. Many studies demonstrate the FCD have great antioxidant activity and immune functions [34,37]. According the previously-mentioned research and our study, FCD could be developed into an anti-oxidant agent, blood lipid-reducing supplement, and we suggest that FCD may be a potential ergogenic aid against abnormal metabolite accumulation and to increase utilization of important fuel source (glucose). In conclusion, our study provides experiment-based evidence to support anti-fatigue function of FCD supplementation and suggests a use for FCD as a potential ergogenic and anti-fatigue agent.

## Supplementary Files

Supplementary File 1
