# Supplementary material for: Fucoidan Supplementation Improves Exercise Performance and Exhibits Anti-Fatigue Action in Mice
Source: Nutrients. 2014 Dec 31;7(1):239–52. doi: 10.3390/nu7010239 (PMC4303836; doi:10.3390/nu7010239)
Supplement: Supplementary File 1 [file nutrients-07-00239-s001.pdf]

CERTIFICATE of ANALYSIS

Product Name: 85% Fucoidan **Batch No.** : 20130104

Production Date: 20130104 **Package** : 1kg/bag

Botanical Name: Laminaria Japonica **Origin of Raw Material**: Laminaria Japonica

Country of Processing: China

Analysis Items	Specifications	Results
Appearance	White Powder	Conformed
Water Solubility	Soluble	Conformed
Viscosity	3~15cp	Conformed
Fucoidan	≥85%	85%
L-Fucose	≥20%	21%
Organic SO_4^{2-}	≥18%	19%
Alginic Acid	≤40%	30%
Raw Protein	≥12%	15%
Lead	≤3ppm	Conformed
Cadmium	≤3ppm	Conformed
Mercury	≤0.1ppm	Conformed
Arsenic	≤2ppm	≤1ppm
Loss on Dry	≤8.0%	4.98%

MICROBIOLOGY

Total Plate Count	<1000 cfu/g	Conformed
Yeast & Mould	<100 cfu/g	Conformed
E. Coli	Not to be Detected	Conformed
Salmonella	Not to be Detected	Conformed

Shelf life: 2 years when properly stored

Usage: Raw Material for production of Dietary Supplements

FDA Registration#13442002850

WEL-BLOOM BIOTECH CORPORATION 逢興生物科技股份有限公司 TEL:3322-5555 新北市中和區立德街143巷25號1F
